# Quantification of Multifunctional Dipeptide YA from Oyster Hydrolysate for Quality Control and Efficacy Evaluation

**DOI:** 10.1155/2018/8437379

**Published:** 2018-09-24

**Authors:** Cheng-Liang Xie, Sang Soo Kang, Ciyong Lu, Yeung Joon Choi

**Affiliations:** ^1^Department of Medical Statistics and Epidemiology, Guangdong Provincial Key Laboratory of Food, Nutrition and Health, Guangdong Engineering Technology Research Center of Nutrition Translation, School of Public Health, Sun Yat-Sen University, Guangzhou 510000, China; ^2^Department of Anatomy and Convergence Medical Science, Institute of Health Sciences, College of Medicine, Gyeongsang National University, Jinju 52727, Republic of Korea; ^3^Department of Seafood Science and Technology/Institute of Marine Industry, Gyeongsang National University, Tongyeong 53064, Republic of Korea

## Abstract

YA is an angiotensin-I-converting enzyme- (ACE-) inhibitory peptide from oyster hydrolysate with antihypertensive activity. Its antioxidant and anti-inflammatory activity were investigated in this study. YA can dose-dependently quench DPPH and ABTS radical and inhibit lipopolysaccharide-induced nitric oxide in RAW 264.7 cells. YA is a multifunctional peptide and was selected as an indicator for quality control and efficacy evaluation of oyster hydrolysate. A practical HPLC/UV assay for YA quantification was developed and validated. It was proved to be accurate and reliable, according to parameters such as specificity, linearity, precision, and accuracy. The quantity results of YA showed that the stage of enzymatic hydrolysis was a critical control point for quality control; the efficacy of oyster hydrolysate can be enhanced after digested in the gastrointestinal tract due to the release of YA by brush border peptidases. Therefore, YA from oyster hydrolysate is a potential bioactive ingredient for functional foods to combat hypertension.

## 1. Introduction

Hypertension, the persistent increase of systolic/diastolic blood pressure over 140/90 mm Hg, respectively, is one of the major risk factors for the development of cardiovascular disease and has become a major public health problem in that it affects about 15-20% of people around the world [1]. In the human body, blood pressure is mainly regulated through renin-angiotensin system (RAS). The angiotensin I-converting enzyme (ACE) is a key enzyme of RAS by which angiotensin I is converted into angiotensin II, a major vasoconstrictor [2]. Excessive levels of angiotensin II result in the development of hypertension and induce the production of oxidative stress and inflammation [3]. Oxidative stress usually results in the overproduction of reactive oxygen species (ROS), which can generate and/or maintain hypertension through inactivating endothelium-derived nitric oxide (NO), promoting smooth muscle cell proliferation, and causing thickening and stiffening of artery [4–6]. Inflammatory cytokines such as interleukin and tumor necrosis factor-*α* (TNF-*α*) can inhibit vasodilator release and result in aggravation of hypertension by downregulating endothelial nitric oxide synthase (eNOS) and damaging endothelium cells [7, 8]. Therefore, multifunctional compounds with a combination of ACE-inhibitory, antioxidant, and anti-inflammatory activity are favorable for use in the prevention and treatment of hypertension due to additional beneficial effects.

Many studies have demonstrated that bioactive peptides from protein hydrolysate exhibit various bioactivities such as those delivering antihypertensive, immunomodulatory, antioxidant, antimicrobial, and anticancer effects [9, 10]. Some bioactive peptides even have multifunctional activities [9]. Considering their safety and the health benefits they offer, great interest has been shown in developing protein hydrolysate containing multifunctional peptides into functional foods for the prevention and/or management of hypertension.

Unlike synthesized chemical drugs, the constituents of bioactive peptides in protein hydrolysates vary greatly depending on hydrolysis conditions [11]. Quantification of bioactive peptides as an indicator for quality control is helpful in maintaining the consistency of composition and the efficacy of the final products [12]. In addition, it has been reported that bioactive peptides in protein hydrolysate can be degraded by digestion enzymes in gastrointestinal tract (GIT), which can result in a decrease of the efficacy [13]. An* in vitro* model that simulates digestion in the GIT has been widely utilized for the evaluation of the stability and efficacy of bioactive compounds in the process of GIT digestion [14]. The quantity of bioactive peptides before and after* in vitro *simulated digestion can help researcher understand the change in the efficacy of protein hydrolysate after digestion in the GIT. Therefore, for quality control and efficacy evaluation, it is necessary to perform a practical analysis assay for bioactive peptides quantification.

In our previous work, an ACE-inhibitory dipeptide YA was identified in the oyster hydrolysate with antihypertensive effects [15]. Here YA was selected as an index substance of oyster hydrolysate for quality control and efficacy evaluation. Considering the effect of oxidative stress and inflammation on hypertension, the object of present study was to investigate the antioxidant and anti-inflammatory activity of YA and develop and validate an RP-HPLC/UV analysis assay for YA quantification in the process procedure of oyster hydrolysate and before and after* in vitro *simulated digestion.

## 2. Materials and Methods

### 2.1. Materials

Frozen oysters were purchased from Daewon Fisheries Co. (Tongyeong, Korea) in October 2014. Protamex, Neutrase 0.8L, and microbial transglutaminase (MTGase) were obtained from Biosis Co. (Busan, Korea) and Ajinomoto Co. (Tokyo, Japan), respectively. Peptide YA, trifluoroacetic acid (TFA), 1,1-diphenyl-2-picrylhydrazyl (DPPH), 2,2'-azinobis-3-ethylbenzothiazoline-6-sulfonic acid (ABTS), and lipopolysaccharide (LPS) from* Escherichia coli* were purchased from Sigma-Aldrich Co. (St. Louis, MO, USA). HPLC grade acetonitrile (ACN) and methanol (MeOH) were obtained from Burdick & Jackson (Muskegon, MI, USA). All other reagents and chemicals were analytical grade. A Watchers C18 column (ODS-AP, 5 *μ*m, 4.6×250 mm) was purchased from Daiso Co. (Tokyo, Japan), and the Hector-A C8 column (5 *μ*m, 4.6× 250 mm) and Hector-A C18 column (5 *μ*m, 4.6×250 mm) were purchased from RS-Tech Co. (Daejeon, Korea).

### 2.2. Scavenging Ability on DPPH Radical

DPPH radical scavenging activity was measured according to the method of You et al. with slight modifications [16]. An aliquot (150 *μ*L) of 0.2 mM DPPH was mixed with 50 *μ*L test sample in 96-well microplates. After incubating at room temperature in the dark for 30 min, the absorbance was measured at 517 nm. The scavenging ability on DPPH radical was calculated as follows:

(1) Scavenging  ability  on  DPPH  radical=ABScontrol−ABSsampleABScontrol∗100

where ABS_control_ is the absorbance of the mixture of DPPH radical and distilled water; ABS_sample_ is the absorbance of the mixture of DPPH radical and the test sample.

### 2.3. Scavenging Ability on ABTS Radicals

ABTS radical scavenging activity was measured according to the method of You et al. with slight modifications [16]. The ABTS radical was produced by mixing 7 mM ABTS stock solution with 2.45 mM potassium persulfate and keeping in the dark at room temperature for 16 h. The ABTS radical solution was diluted by 50 mM phosphate buffer saline (pH 7.4) to an absorbance of 0.70 ± 0.02 at 734 nm. Then, 190 *μ*L of diluted ABTS radical solution was mixed with 10 *μ*L of test samples in 96-well microplates. After the mixture was incubated at room temperature in the dark for 6 min, the absorbance was measured at 734 nm. The scavenging ability on ABTS radical was calculated as follows:

(2) Scavenging  ability  on  ABTS  radical=ABScontrol−ABSsampleABScontrol∗100

where ABS_control_ is the absorbance of the mixture of ABTS radical and distilled water; ABS_sample_ is the absorbance of the mixture of ABTS radical and the test sample.

### 2.4. Nitric Oxide- (NO-) Suppressing Activity

NO-suppressing activity was measured according to the method of Tsai et al. [17]. The mouse macrophage cell line RAW 264.7 was obtained from the American Type Culture Collection (Manassas, VA, USA) and cultured in Dulbecco's Modified Eagle's Medium (DMEM) supplemented with 10% fetal bovine serum (FBS) and 1% penicillin-streptomycin (Gibco, NY, USA) at 37°C in a humidified atmosphere containing 5% CO_2_. After subculture, RAW 264.7 cells were seeded in 24-well plates (5×10^5^ cells/well) in DMEM and incubated for 12 h. The adherent cells were treated with a test sample together with LPS (1 *μ*g/mL) for 24 h. Following incubation for 24 h, the medium was collected and medium nitrite concentration was measured by Griess reagent as an indicator of NO production. A NaNO_2_ standard curve was used to calculate the nitrite concentration for control and samples. The NO-suppressing activity was expressed as the percent of control value, which was the nitrite concentration from only LPS-activated RAW 264.7.

### 2.5. Development of RP-HPLC Analysis

The UV-VIS spectrophotometer (OPTIZEN POP, Mecasys Co., Korea) was used to record the absorption spectrum in the range of 200-400 nm of peptide YA solution (0.1 mg/mL). RP-HPLC analysis was performed using a HPLC system (Shimadzu Co., Kyoto, Japan) equipped with LC-20AD pump, SIL-20A autosampler, SPD-20A detector, and temperature control module. This study employed three different RP-HPLC columns: Watchers C18, Hector-A C18, and Hector-A C8. The optimized separation conditions with Watchers C18 column consisted of 0.1% TFA in water (A) and 0.1% TFA in ACN (B) with the increase B from 0.8-5.0% B in 40 min at the flow rate of 1 mL/min. The injection volume was 5 *μ*L. UV detection was performed at 273 nm. Shimadzu LabSolutions/ LC solution software was used to calculate the column efficiency parameters.

### 2.6. Method Validation

Validation was performed based on the International Conference on Harmonization Guidance for the Validation of Analytic Method [18]. The following characteristics were evaluated: specificity, linearity, sensitivity, accuracy, and precision. Specificity was evaluated by spiking synthetic YA in the sample with equal volume. The YA peak was identified by comparison of data from retention time of HPLC of the synthetic YA. A calibration curve was obtained by plotting the peak area against six concentrations of the synthetic YA solution (n=3) in the concentration range of 5-100 *μ*g/mL and its linearity of regression line was evaluated. The recovery of YA was calculated as the percentage of the measured concentration to the true concentration of the YA standard solution at six levels. Sensitivity was determined by evaluating of the limit of detection (LOD) and quantification (LOQ) as follows:

(3) LOD=3SσbLOQ=10Sσb

where S_*σ*_ is the residual standard deviation and b is the slope of the calibration curve.

Intraday and interday accuracy were determined by the percentage bias from the true concentration using nine determinations (three concentrations/three replicates each). Precision was determined by repeatability (intraday) and intermediate precision (interday). Repeatability refers to the precision under the same operating conditions over a short interval of time (in the same day). Intermediate precision was assessed on three different days. Accuracy was expressed by bias%. Repeatability and intermediate precision was expressed by relative standard derivation (RSD%).

### 2.7. Process Procedure of Oyster Hydrolysate

Oyster hydrolysate was prepared according to the previous method with slight modification [15]. Briefly, the minced oysters were cross-linked by 1% MTGase to the weight of the oyster at 30°C for 1 h, and then they were hydrolyzed by a two-step hydrolysis process with 1% Protamex and Neutrase to the weight of the oyster for 1 h at 40 and 50°C, respectively. The hydrolysis was terminated at 100°C for 30 min. The hydrolysate was filtered with a cake filter (200 mesh) and concentrated at 100°C by a concentrator (HS-1000 L, Hansung F&C Co., Incheon, Korea) until about 30 Brix and then dried by a spray dryer (20K, Yoojin Tech Co., Pyeongtaek, Korea) in a cocurrent air flow mode. The inlet and outer temperatures were set at 120°C and 90°C, respectively. At each stage, a sample was taken for YA quantification.

### 2.8. In Vitro Simulated Digestion

The* in vitro* simulated digestion was completed according to the method of Vercruysse et al. with slight modification [14]. Pepsin (Product No. P7000), trypsin (Product No. T9201), *α*-chymotrypsin (Product No. C7762), and carboxypeptidase A (Product No. SAE0046) were purchased from Sigma-Aldrich Corporation (Saint Louis, MO, USA). The ratio of enzyme to substrate (E/S) is defined as the ratio of the concentration between enzyme and substrate. Pepsin was added (E/S, 1:100) at pH 2, and the mixture was incubated for 2 h at 37°C to simulate digestion in the stomach. Trypsin and *α*-chymotrypsin were added (1:1; E/S, 1/100) at pH 6.5 and incubated for 2.5 h at 37°C to simulate the digestion phase in the small intestine. Carboxypeptidase A was added (E/S, 1/1000) at pH 7.5 and incubated for 2 h at 37°C to simulate an attack by peptidases from intestinal brush border. Finally, the digestion process was terminated by adjusting the mixture to pH 4 with 1M HCl. After each digestion phase, the digest mixture was taken and the protease was inactivated in a boiling water bath for 15 min. The YA content in the digest mixture was determined and expressed as a percentage compared with that of the oyster hydrolysate without treatment by protease, which was used as a control.

### 2.9. Statistical Analysis

Data were expressed as the mean and standard deviation of at least triplicate for each sample. One-way analyses of variance (ANOVA) were used to analyze the between-group differences. After this, GraphPad Prism 5 software (GraphPad Software Inc., La Jolla, CA) was used to carry out Tukey's HSD test.

## 3. Results and Discussion

### 3.1. Antioxidant and Anti-Inflammatory Activity of YA

**Figure 1:**
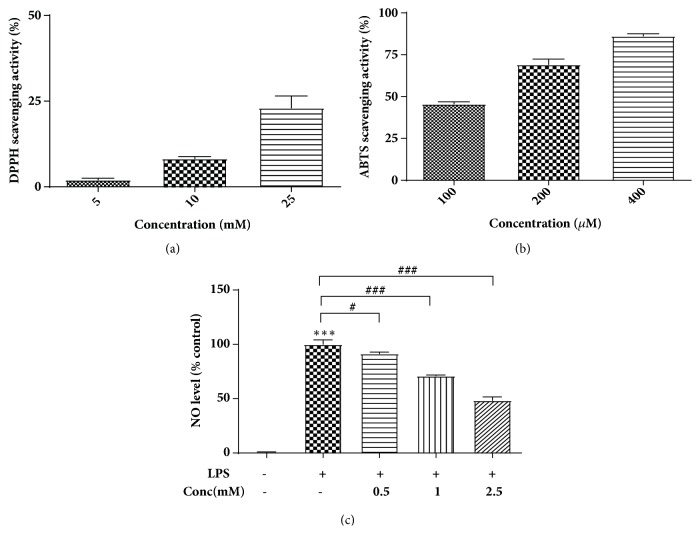
The scavenge activity of DPPH (a) and ABTS (b) radical and nitrite oxide- (NO-) suppressing activity of YA (c). DPPH, 1,1-diphenyl-2-picrylhydrazyl; ABTS, 2,2'-azinobis-3-ethylbenzothiazoline-6-sulfonic acid; *∗∗∗p *< 0.001 versus without lipopolysaccharides (LPS) treatment; #* p*<0.05 and ###*p *< 0.001 versus only LPS treatment.

ABTS and DPPH radical scavenging assay were used to determine the antioxidant activity of YA. The ABTS radical scavenging assay is based on decolorization of blue/green ABTS radical, whereas the DPPH assay is based on the reduction of purple DPPH to a yellow colored 1,1-diphenyl-2-picryl hydrazine in the presence of antioxidant. Both of these have been widely used to evaluate the antioxidant activity of compounds [19]. Our results revealed that YA exhibits a dose-dependent DPPH and ABTS radical scavenging activity, while the scavenging capacity of ABTS radical was much higher than that of DPPH radical (Figures 1(a) and 1(b)). This can be explained by that YA and ABTS radical are water soluble but DPPH radical is lipid soluble, so YA showed much stronger antioxidant activities in the aqueous system than that in the lipid soluble system [20]. According to the structure of YA, it contained Tyr residue, which was generally accepted as antioxidant and, to some extent, can be attributed to antioxidant activity of YA [21]. To compare the antioxidant activity of YA with Tyr, the ABTS scavenging ability of Tyr also was investigated (Supplementary Figure S1). The 50% scavenger concentration of YA and Tyr for ABTS radical was 116 and 267 *μ*M, respectively, which showed YA had much stronger activity than Tyr. Consistent with our results, it has been reported that dipeptides consisting of Tyr at the N-terminus showed higher antioxidative activities than the constituent amino acid in an aqueous system [22].

The anti-inflammatory effect of YA was evaluated by NO-suppression activity in LPS-stimulated RAW 264.7 cells. LPS is an endotoxin from outer membrane of gram-negative bacteria and can stimulate macrophages to produce inflammation-related mediators such as NO [23]. The model of LPS-induced inflammation in RAW 264.7 has been widely utilized for screening of anti-inflammatory drugs [24]. In order to avoid cytotoxic effects on NO production, first we investigated the effect of YA on cell viability of RAW 264.7 cells. In our results, no cell toxicity was observed at the test concentration between 10 and 2,500 *μ*M (Supplementary Figure S2). Therefore, the RAW 264.7 cells were treated with LPS combined with different concentration of YA and NO levels were determined (Figure 1(c)). Results showed that LPS-stimulated RAW 264.7 cells more significantly increase the concentration of NO than do unstimulated RAW 264.7 cells in the culture medium, whereas the levels of LPS-induced NO were dose-dependently inhibited by YA, reducing by 52.6% when compared with that of only LPS-stimulated RAW264.7 cells at the concentration of 2.5 mM. Amino acid Tyr also showed NO-suppressing activity (reduced by 20.8%) in high concentration (2.5 mM), but it was much lower than that of YA (Supplementary Figure S3). It has been reported that overproduction of NO can result in pathogenesis of hypertension [25]. YA can ameliorate hypertension by inhibition the production of NO.

Besides oyster hydrolysate, YA was also identified in hydrolyzed maize protein and showed the activities of enhancing the lifespan and healthspan of* Caenorhabditis elegans* by decreasing free radical and upregulating stress-resistance-related proteins* in vivo* [26]. YA has much higher solubility than amino acid Tyr, which can enhance its bioavailability* in vivo* [26, 27]. Therefore, multifunctional YA may have potential for the prevention and/or management of hypertension.

### 3.2. Development of RP-HPLC Method for YA Quantification

**Figure 2:**
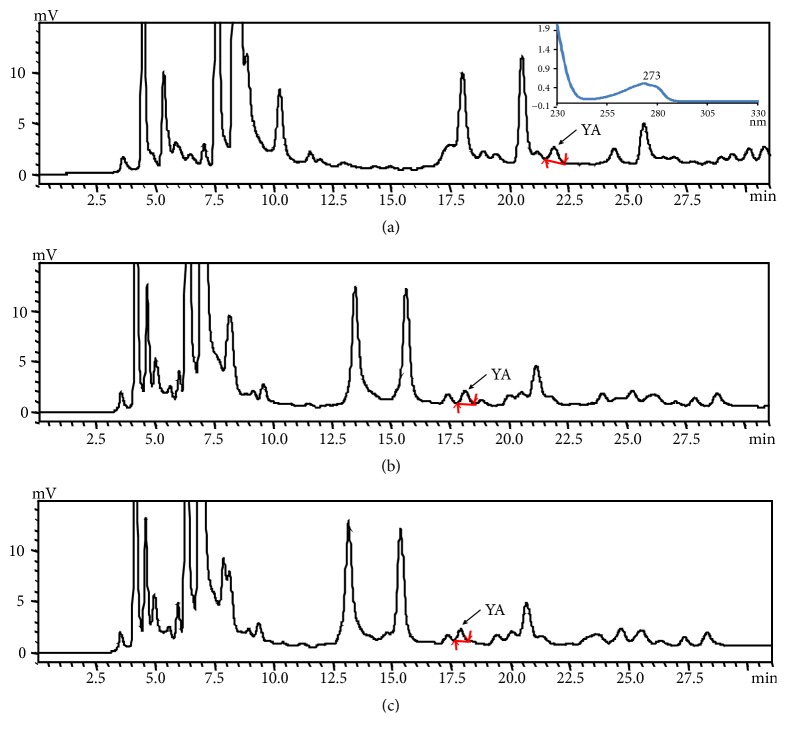
The UV spectrum and HPLC chromatogram of the oyster hydrolysate solution with different initial concentration and 5% final concentration of organic solvent for 40 min in the gradient model: (a) 0.1% TFA; (b) 0.1% TFA/ 0.8% ACN; (c) 0.1% TFA/1% ACN. TFA, trifluoroacetic acid; ACN, acetonitrile.

In order to obtain better separation of YA in chromatograms, the wavelength, column, and mobile phase were investigated in this study. The ideal detection wavelength should have the greatest sensitivity with little noise. YA containing Tyr residues had the maximum absorption at 273 nm (Figure 2). It has been reported the UV absorption of peptide arising from peptide backbone is between 190 and 230 nm, if aromatic amino acid residues are not present in the peptides [28]. The specific detection wavelength of 273 nm could maintain the greatest sensitivity for YA and avoid the interference of nonaromatic peptides. For selection of effective columns and mobile phases, three different RP columns were tested by the isocratic elution with 3% ACN and 3% MeOH, respectively. It is well known that, due to their high optical transparency in the detection wavelengths used for peptide analysis, ACN and MeOH are most commonly used in the mobile phase in RP-HPLC [29]. At the same elution condition of 3% ACN, the theoretical plate number (T.plate) of Watchers C18 column (T.plate, 8477) was higher than that of the other two columns (T.plate, Hector-A C8, 3468.2; Hector C18, 3819.6) (Table 1), which suggested that Watchers C18 column had the highest resolution ability. In the case of 3% MeOH, there was a little increase of T.plate of Watchers C18 columns; meanwhile the half-peak width became much broader compared to that of 3% ACN (Table 1). The Watchers C18 and organic solvent ACN were chosen as column and mobile phase, respectively, for YA analysis based on the results of the T.plate number and half-peak width. A low concentration of TFA (0.1%) was generally added to the mobile phase to suppress the ionization of peptides for preventing polar interaction between the peptide and active site on the column, minimizing peak broadening, and improving the separation selectivity [30].

More frequently than isocratic model, gradient elution in the analysis of peptide was commonly chosen due to high efficiency in the separation of analytes with a wide range of polarity. For gradient elution, the separation efficiency of peptides was significantly affected by the parameters such as gradient steepness and the initial and final concentration of organic solvent [31]. Isocratic elution with different concentration of ACN was performed first in order to determine parameter values of gradient elution. The retention time was greatly decreased from 28.821 to 9.707 min with the increased concentration of ACN from 0 to 3% (Table 1). The retention time of YA was modified significantly by the concentration of ACN in the mobile phase. By a trial and error increase of B concentration from 0.8 to 5% in 40 min, a binary gradient with mobile phase A (0.1% TFA in water) and B (0.1% TFA in ACN) was developed (Figure 2). In the optimization condition, high water content in the mobile phase was used, which demanded the RP column was same as Watchers ODS-AP C18 column to be treated by high coverage and exhaustive end-capping to avoid phase collapse from the self-association of long alkyl-chains of the hydrophobic bonded phase in the high water content of the mobile phase [32].

### 3.3. Validation of RP-HPLC Method for YA Quantification

**Figure 3:**
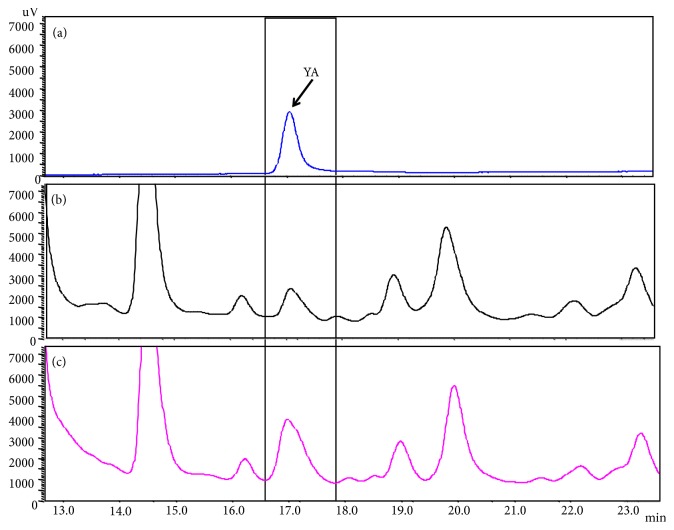
HPLC chromatograms of YA (a), the oyster hydrolysate solution (b), and the oyster hydrolysate solution spiked with YA (c).

Specificity is one of the most important characteristics of an analysis method. When the criteria for specificity are not met, this often indicates that the method is not sufficiently developed [33]. YA was well separated from the adjacent peaks and was identified by comparison of the retention time and cochromatography with standard YA (Figure 3). The mass of YA was 252.7 by mass spectrometer, which was well matched with that of standard YA of 252.6 and the theoretical calculation value of 252.3 (Supplementary Figure S4). The plot of peak area versus YA concentration showed a good linearity (r^2^=0.999) in the range of 5-100 *μ*g/mL by the developed analysis method, and its recoveries were 99.4-100.3% (Table 2). The LOD and LOQ for the quantification of YA were calculated as 0.5 and 1.7 *μ*g/mL, respectively. The LOD value in our study was lower than that of VPP (4 *μ*g/mL) and IPP (2 *μ*g /mL) in the fermented milk [34]. Moreover, accuracy ranged from -4.1 to 0.9% and precision ranged from 0.2-4.8% (Table 3). All the results of accuracy and precision were within the acceptance criteria of 5% [35].

### 3.4. YA Content in the Process Procedure

The process procedure of oyster hydrolysate goes through a serial of processing stages from frozen oyster to final products of oyster hydrolysate powder. The quantification of YA for each stage is necessary for the selection of critical control points and quality control. As shown in Table 4, YA cannot be detected before enzymatic hydrolysis. After two-step sequential hydrolysis with Protamex and Neutrase, the YA increased markedly, which was in agreement with the results of van der Ven that a bioactive peptide can be released from a parent protein through enzymatic hydrolysis [11]. After remaining process procedure of inactivating, filter, concentration, and spray-drying, the YA content in the final products decreased slightly more than in the stage of two-step sequential hydrolysis. Among all the process stage, the stage of sequential hydrolysis was very important in terms of which YA was produced and how much the efficacy of the final oyster hydrolysate was decided. Therefore, the stage of hydrolysis can be viewed as a critical control point for quality control of oyster hydrolysate.

### 3.5. The Change of YA Content in Simulated Digestion

**Figure 4:**
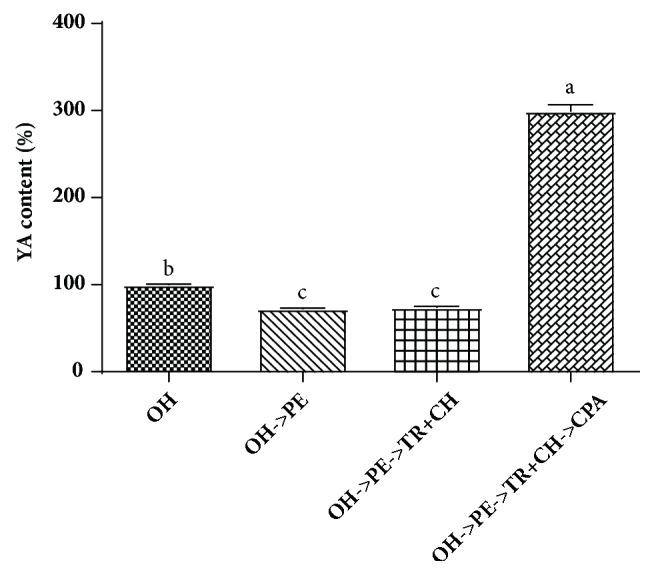
YA content after treatment with pepsin, trypsin and *α*-chymotrypsin, and carboxypeptidase A compared with that of control (without enzyme treatment). OH, oyster hydrolysate; PE, pepsin; TR, trypsin; CH, *α*-chymotrypsin; CPA, carboxypeptidase A. The labels with different letters above each bar denote statistical significance (*p* < 0.05).

After oral administration, bioactive peptides from protein hydrolysate may reach circulation in human blood system by intestinal absorption, thereby exerting their biological activity* in vivo* [36]. However, bioactive peptides are liable to be degraded by proteases in the GIT, causing a decrease or loss in their efficacy [13]. The quantity of bioactive peptide via* in vitro* simulated digestion may constitute a better indicator for the evaluation of efficacy* in vivo*. The content of YA for each digestion stage was shown in Figure 4. After oral administration, oyster hydrolysate was, first, digested in stomach. In the stage of simulated stomach liquid, YA decreased by 27.8%. It has been reported that gastric fluids degraded peptides mainly by low gastric pH, altering the ionization of amino acids residues and pepsin, cleaving peptide bonds within a peptide chain between hydrophobic, preferably, aromatic amino acids [37]. Subsequently, digestion continued in the small intestine. At this stage of simulated intestinal fluids, no significant change was observed for YA content, which can be attributed to the size and the cleavage point of trypsin and chymotrypsin. YA has small size, so it can be completely stable in the fluid of small intestine [37]. Moreover, the cleavage point of trypsin generally sites the C-terminal side of lysine and arginine, and chymotrypsin prefers cleavage on the carboxyl side of aromatic amino acid residues, phenylalanine, tryptophan, and tyrosine [38]. Finally, the digested oyster hydrolysate reached the intestinal brush border, where absorption take place. After being digested by carboxypeptidase A, the YA content increased by 199.4%. Carboxypeptidase is one class of brush border peptidases. Among carboxypeptidases, carboxypeptidase A has a stronger preference for those amino acids containing aromatic or branched hydrocarbon chains and cleave these amino acid residues from the COOH terminus, to yield free amino acids or smaller di- or/and tripeptides [39]. Overall, based on the quantity of YA in simulated digestion, the efficacy of the oyster hydrolysate can partly loss due to the degradation by gastric fluid but can be enhanced markedly in the role of brush border peptidases. However, to confirm the results, it is necessary to examine the pharmacokinetics of YA in oyster hydrolysate* in vivo* at some time in the future.

## 4. Conclusions

YA, an ACE-inhibitory peptide from oyster hydrolysate, had antioxidant and anti-inflammatory activity. A practical HPLC/UV method for YA quantification was first developed and validated. Based on the quantity of YA in each process procedure of oyster hydrolysate, YA were mainly produced in the stage of enzymatic hydrolysis, which can be set as a critical control point for quality control. According to the results from the simulated digestion, during GIT digestion, a loss in the efficacy of oyster hydrolysate will not happen; in contrast, the efficacy will be enhanced due to the increase of YA through releasing from parent peptides by brush border peptidases. Therefore, oyster hydrolysate containing multifunctional YA has a potential for use as the ingredient in functional food for protection against hypertension.

## Figures and Tables

**Table 1:** The values of the column efficiency parameters with the different mobile phase in the isocratic model.

Column	Mobile phase	R_t_ (min)	W_50%_ (min)	T.plate
Hector-A C8	0.1%TFA/3% ACN	3.473	0.132	3468.2
Hector-A C18	0.1% TFA/3% ACN	5.145	0.191	3819.6
Watchers C18	0.1% TFA/3% MeOH	13.706	0.331	9732.6
Watchers C18	0.1% TFA/3% ACN	9.707	0.244	8477.0
Watchers C18	0.1% TFA/2% ACN	14.427	-	-
Watchers C18	0.1% TFA/1% ACN	20.925	-	-
Watchers C18	0.1%TFA	28.821	-	-

R_t_, retention time; W_50%_, half-peak width; T.plate, theoretical plate number; TFA, trifluoroacetic acid; ACN, acetonitrile; MeOH, methanol; -, no calculation.

**Table 2:** Concentration, area, and recoveries of synthetic YA solution.

Concentration (*μ*g/mL)	Area	Recovery (%)
5	6,760±54	99.4±0.8
10	13,577±82	99.8±0.6
20	27,292±77	100.3±0.3
40	54,349±51	99.9±0.1
80	108,469±295	99.7±0.3
100	136,270±129	100.2±0.1

**Table 3:** Accuracy and precision of the RP-HPLC method in both intra- and interday.

		Concentration (*μ*g/mL)		
		24.1	34.1	54.1
Intraday	Day 1			
	Mean	24.1	34.4	54.0
	SD^a^	0.6	0.1	0.9
	RSD%^b^	2.6	0.2	1.7
	Bias%^c^	-0.5	0.9	-0.2
	Day 2			
	Mean	23.1	33.6	52.0
	SD^a^	1.0	0.1	0.4
	RSD%^b^	4.2	0.4	0.8
	Bias%^c^	-4.1	-1.5	-3.9
	Day 3			
	Mean	23.2	32.9	53.7
	SD^a^	1.1	0.8	2.6
	RSD%^b^	4.6	2.4	4.8
	Bias%^c^	-3.6	-3.4	-0.7

Interday	Mean	23.6	33.6	53.2
	SD^a^	0.9	0.8	1.7
	RSD%^b^	3.6	2.2	3.1
	Bias%^c^	-2.2	-1.4	-1.6

^a^SD, standard deviation; ^b^RSD, relative standard deviation; RSD%=(standard deviation/mean)×100; ^c^Bias%= (measured concentration-true concentration)/true concentration×100.

**Table 4:** The YA content in processing stages of the oyster hydrolysate (*μ*g/g, dry weight).

Process stages	Content
Frozen oyster	ND
Blanching and mincing	ND
Solubilization with water and cross-linking by MTGase	ND
Hydrolysis with Protamex and Neutrase	44.4±2.1
Inactivating the enzyme	36.0±0.9
Filter and concentration	36.7±3.2
Spray-drying	35.7±0.9

ND, not detected.

## Data Availability

The Excel data used to support the findings of this study have been deposited in the figshare repository (DOI 10.6084/m9.figshare.6354020).

## Supplementary Materials

Supplementary Materials Supplementary material contains some additional data for this paper. Figure S1: ABTS scavenging activity of Tyr. Figure S2: cell viability of RAW 264.7 cells treated by different concentration of YA. Figure S3: cell viability and nitride oxide-suppression activity on RAW 264.7 cells treated by different concentration of Tyr. Figure S4: the mass spectra of synthesized YA and the YA fraction separated from the oyster hydrolysate by AKTA purifier LC system.
