# Supplementary material for: Quantification of Multifunctional Dipeptide YA from Oyster Hydrolysate for Quality Control and Efficacy Evaluation
Source: Biomed Res Int. 2018 Sep 24;2018:8437379. doi: 10.1155/2018/8437379 (PMC6174814; doi:10.1155/2018/8437379)
Supplement: Supplementary Materials — Supplementary material contains some additional data for this paper. Figure S1: ABTS scavenging activity of Tyr. Figure S2: cell viability of RAW 264.7 cells treated by different concentration of YA. Figure S3: cell viability and nitride oxide-suppression activity on RAW 264.7 cells treated by different concentration of Tyr. Figure S4: the mass spectra of synthesized YA and the YA fraction separated from the oyster hydrolysate by AKTA purifier LC system. [file 8437379.f1.docx]

Supplementary Fig. S1

Fig.S1 ABTS scavenging activity of Tyr

Supplementary Fig. S2

Fig.S2 Cell viability of RAW 264.7 cells treated by different concentration of YA.

Supplementary Fig. S3

**B**

Fig. S3 Cell viability (A) and nitride oxide (NO)-suppression activity (B) on RAW 264.7 cells treated by different concentration of Tyr. ****P* < 0.001 versus no LPS treatment; ###*P* < 0.001 versus LPS treatment only.

Supplementary Fig. S4


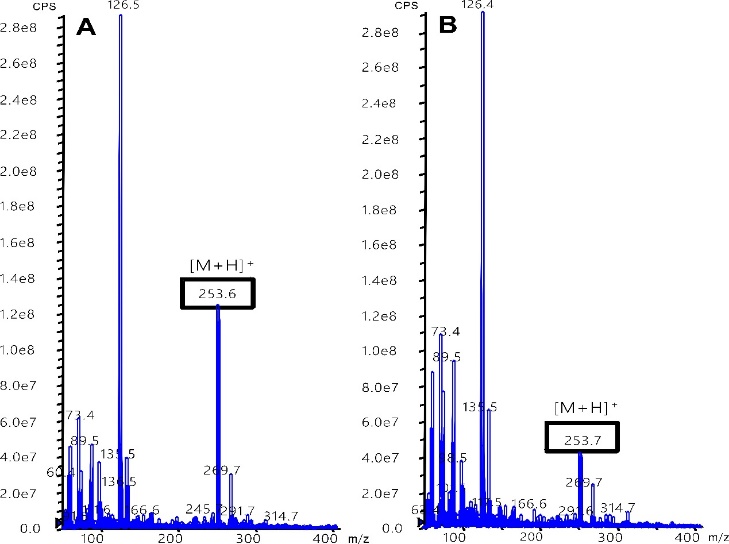


Fig. S4 The mass spectra of the standard YA (A) and the YA separated from the oyster hydrolysate by AKTA purifier LC system (B); CPS, counts per second.

**Analysis condition:**

The oyster hydrolysate solution was fractionated using an AKTA purifier LC system (GE Healthcare, Uppsala, Sweden) with Watchers C18 column (ODS-AP, 5 μm, 4.6×250 mm, Daiso Co., Tokyo, Japan). UV detection was performed at 273 nm. About 20 μL of extract was loaded onto the column. Gradient elution was carried out with 0.1% TFA (A) and 0.1% TFA in ACN (B) with the increase B at a constant flow rate of 1.0 mL/min. The linear gradient elution program was 0.8-5.0% B in 40 min. Meanwhile, each 1 mL was fractionated. The YA fraction was collected by comparison of retention time with standard, and the mass of YA was determined by the mass spectrometer. For MS, the residue after rotary evaporation was reconstituted with 0.1% formic acid/50% ACN. The sample was injected by a Harvard syringe pump at flow rate of 10 μL/min and detected mass in the positive ion and Q1M1 scan using a QTRAP mass spectrometer (API 3200, AB SCIEX, Framington, MA, USA).
